# On estimation for accelerated failure time models with small or rare event survival data

**DOI:** 10.1186/s12874-022-01638-1

**Published:** 2022-06-11

**Authors:** Tasneem Fatima Alam, M. Shafiqur Rahman, Wasimul Bari

**Affiliations:** 1 Institute of Statistical Research and Training, University of Dhaka, Dhaka, Bangladesh; 2 Department of Statistics, University of Dhaka, Dhaka, Bangladesh

**Keywords:** Bias reduction, Monotone likelihood, Jeffreys prior, Log-location-scale family

## Abstract

**Background:**

Separation or monotone likelihood may exist in fitting process of the accelerated failure time (AFT) model using maximum likelihood approach when sample size is small and/or rate of censoring is high (rare event) or there is at least one strong covariate in the model, resulting in infinite estimates of at least one regression coefficient.

**Methods:**

This paper investigated the properties of the maximum likelihood estimator (MLE) of the regression parameters of the AFT models for small sample and/or rare-event situation and addressed the problems by introducing a penalized likelihood approach. The penalized likelihood function and the corresponding score equation is derived by adding a penalty term to the existing likelihood function, which was originally proposed by Firth (Biometrika, 1993) for the exponential family models. Further, a post-hoc adjustment of intercept and scale parameters is discussed keeping them out of penalization to ensure accurate prediction of survival probability. The penalized method was illustrated for the widely used log-location-scale family models such as Weibull, Log-normal and Log-logistic distributions and compared the models and methods uisng an extensive simulation study.

**Results:**

The simulation study, performed separately for each of the log-location-scale models, showed that Firth’s penalized likelihood succeeded to solve the problem of separation and achieve convergence, providing finite estimates of the regression coefficients, which are not often possible by the MLE. Furthermore, the proposed penalized method showed substantial improvement over MLE by providing smaller amount of bias, mean squared error (MSE), narrower confidence interval and reasonably accurate prediction of survival probabilities. The methods are illustrated using prostate cancer data with existence of separation, and results supported the simulation findings.

**Conclusion:**

When sample size is small (≤ 50) or event is rare (i.e., censoring proportion is high) and/or there is any evidence of separation in the data, we recommend to use Firth’s penalized likelihood method for fitting AFT model.

**Supplementary Information:**

The online version contains supplementary material available at (10.1186/s12874-022-01638-1).

## Background

It is now well established in generalized linear model literature that maximum likelihood estimation provides consistent estimates of the regression parameters when sample size is large. However, it may fail to provide a finite or unbiased estimate for at least one regression parameter of the model if sample size is small [10]. The small-sample consequences arise frequently and become worse if there exists separation in the data [12]. The problem of separation or monotone likelihood, first introduced by [1] in binary regression model is a special condition in a dataset which breakdown the standard maximum likelihood method during fitting process of the model resulting in non-existence (or infinite values) of the maximum likelihood estimates. The situation of separation or monotone likelihood may arise when dataset is small in size as well as if event or non-event of interest can be separated mostly by a binary covariate or a linear combination of several covariates [1, 3, 12, 13]. In the presence of separation, the traditional maximum likelihood estimation approach may provide highly biased, even infinite estimates for the regression coefficients of one or more covariates and hence provide Wald confidence intervals of infinite width [11, 12, 17, 25]. A number of studies discussed finite sample bias correction in the maximum likelihood estimate of the regression coefficient and provided solution to separation [6, 7, 18, 24]. To address the problem of separation and infinite estimates, Firth’s preventive method [10] is widely used in statistical inference as it eliminates the first order term *O*(*n*^−1^) in the asymptotic bias of the estimated parameters by solving the modified estimating equation resulted from the addition of a Jeffrey’s invariant prior based penalty term to the original likelihood function. The performance of Firth’s approach in proving the bias reduced estimates and resolving the problem of separation has been demonstrated for the logistic regression model [13] and other models under exponential family of the distributions [4, 15, 19].

The separation or monotone likelihood issue in the context of survival data were first introduced by [12], where it was argued that the pattern of such problem and its consequences in survival data is very similar as in the binary data. However, one may visualize it differently for survival data context. For example, for a single covariate, this occurs when, at each failure time, the covariate value for the failed subjects is the largest of all covariate values in the risk set at that time, or when it is always the smallest. It also occurs when the same is true for a linear combination of covariates. As discussed by Heinze and Schemper, the problem of separation is likely to occur if the sample size is small, percentage of censoring is high, and/or there are one or more strong covariates, particularly binary covariates. The chance of separation increases with the degree of imbalance in the distribution of binary covariate. However, the separation is rarely occur with the continuous covariate and uncensored data. Whatever the reasons for occurring separation in survival data, the situation is by no means negligible as it creates several consequences. To overcome the problems due to small sample, or rare events, or separation in analyzing survival data, [12] suggested a penalized likelihood function for semi-parametric Cox proportional hazard model [8] by incorporating the Firth’s bias preventive principle into the partial likelihood function. However, an appropriately fitted parametric survival model with correctly specified distribution always yields consistent and more efficient estimates of the parameters of interest than the estimates obtained from a semi-parametric model [9, 20] and have intuitive interpretation through a direct connection with the failure time. Moreover, the estimation technique under a parametric model is computationally more flexible and provides precise estimates since both the survival and censoring times are used directly to construct the likelihood function. To provide bias corrected estimate in the small sample situation, [21] applied Firth’s penalized approach to exponential survival model, which, however, has limited use in practice because of inapplicability of constant failure rate assumption in most real world applications. Therefore, under the parametric framework, it is obvious to focus on widely used accelerated failure time (AFT) model, which is a general framework of a range of parametric survival models under log-location scale family of distributions. However, the performance of maximum likelihood estimation technique to estimate the parameters of the AFT models has not been investigated yet when sample size is small or event of interest is rare or if there exists separation in the survival data. In this study, an attempt has been made to examine performance of maximum likelihood estimators in such situations through conducting extensive simulation studies. To address the problems in the MLEs, this paper proposed penalized likelihood estimation for AFT survival models by incorporating the Firth’s penalty term to the original likelihood function, as motivated by [12]. The empirical performance of the newly proposed approach was studied through simulations, where penalized likelihood function was optimized by using quasi-Newton-algorithm.

Though the main purpose of the AFT survival models is to examine how the covariates influence the survival times, estimate of scale parameter is of interest as estimates of both regression and scale parameters are required to predict the survival quantities such as survival probabilities and hazard functions. However, imposing Jeffrey’s prior for penalization may result in further shrinkage of the estimate of scale and intercept parameters, which may lead to biased estimates of survival quantities. This is motivated by the recent study of [22] who identified that Firth’s penalization for logistic regression resulted in inaccurate prediction of overall probability and proposed a correction in the intercept term to ensure accurate prediction. Following the first modification of Firth’s procedure suggested by [22] in logistic regression, this paper also proposed a post-hoc adjustment of the intercept and scale parameters estimated by the penalized likelihood method in the AFT model by keeping them out of penalization.

The paper is organized as follows. “Methodology” section describes the methodology starting with a brief discussion on AFT model and maximum likelihood estimation procedure, which is followed by the application of Firth’s principle to derive the penalized likelihood function for AFT model and optimization procedure and ended by the post-hoc adjustment of the scale parameters. Comprehensive simulation studies conducted under different scenarios to compare the performance of estimates obtained through the maximum likelihood and penalized likelihood functions are given in “Simulation study” section. An illustration of the proposed methods using data on prostate cancer patients [5] is discussed in “Illustration using prostate cancer data” section. This paper concludes with a brief discussion on findings, limitations, and further scope of this study in “Discussion” section.

## Methodology

### AFT model

Let us consider a censored random sample containing data (*y*_*i*_,*δ*_*i*_,***x***_*i*_),*i*=1,…,*n*, where *y*_*i*_= log(*t*_*i*_) is a log-lifetime or log-censoring-time if the censoring indicator *δ*_*i*_=1 or *δ*_*i*_=0, respectively and ***x***_*i*_=(1,*x*_*i*1_,…,*x*_*ir*_,…,*x*_*ip*_)^*T*^ is a (*p*+1)-dimensional vector of covariates. The location-scale family model describes the relationship between the survivor function *S*(*y*_*i*_|***x***_*i*_) and a set of covariates ***x*** as

$$\begin{array}{*{20}l} S(y_{i}|\boldsymbol{x}_{i})=S_{0}\Big(\frac{y_{i}-u(\boldsymbol{x}_{i}\boldsymbol{\beta})}{b}\Big), \;\; -\infty< y<+\infty \end{array} $$

where *S*_0_(*z*) is the survivor function of a standardized random variable, *u*(***x***_*i*_,***β***)=***β***^*T*^***x***_*i*_ denotes location parameter and *b* the scale parameter, and ***β***=(*β*_0_,*β*_1_,…,*β*_*r*_,…,*β*_*p*_)^*T*^ is a vector of regression coefficients [16]. The model can also be written in the following form to describe the relationship between covariate and log-survival time:

1 $$\begin{array}{*{20}l} y_{i}=u(\boldsymbol{x}_{i}\boldsymbol{\beta})+bZ_{i}, \end{array} $$

where *Z*_*i*_ is a random variable with a standard distribution in (−*∞*,*∞*). The covariate effectively alters the log-time scale in additive form (or original time-scale in multiplicative form) and hence the model is referred to as accelerated failure time (AFT) model. The above equation represents as a family of models for which *Z* belong to a standard location-scale family of distributions (i.e., extreme value, logistic, normal distribution) while the survival time *T* belonging to the log-location scale family of distributions (i.e., Weibull, log-logistic, log-normal), respectively.

### Maximum likelihood estimation for AFT model

The maximum likelihood method is commonly used to estimate the parameter of the model given in Eq. (1). Setting *z*_*i*_=(*y*_*i*_−*u*_*i*_)/*b* with p.d.f $f_{0}(z)=-S^{'}_{0}(z)$; *u*_*i*_=*u*(***x***_***i***_;***β***) and $m=\sum _{i=1}^{n}\delta _{i}$, the log-likelihood function for the location-scale family model (1) can be written as:

2 $$ \begin{aligned} \ell(\boldsymbol{\beta},b)=-m\log b+\sum_{i=1}^{n}\left[\delta_{i}\log f_{0}(z_{i})+(1-\delta_{i})\log S_{0}(z_{i})\right]. \end{aligned}  $$

Let ***θ***=(***β***^*T*^,*b*)^*T*^ be the (*p*+2)-dimensional vector of parameters. The maximum likelihood estimate of ***θ*** is the solution of the estimating equations *U*(***β***,*b*)=***0*** simultaneously, where *U*(***β***,*b*) is the score function defined as

$$\begin{aligned} U(\boldsymbol{\beta},b)=&\partial \ell(\boldsymbol{\theta})/\partial \boldsymbol{\theta}\\=&[\!U_{0}(\boldsymbol{\beta},b),\! U_{1}\!(\boldsymbol{\beta},\!b), \dots\!, U_{r}(\boldsymbol{\beta},b),\ldots\!, U_{p}(\boldsymbol{\beta},b), U_{b}(\boldsymbol{\beta},b)]^{T}\!. \end{aligned} $$

If ***X*** is an *n*×(*p*+1) matrix having rows $\boldsymbol {x}_{i}^{T}=(1, x_{i1}, \ldots, x_{ir}, \ldots, x_{ip})$, then *∂z*_*i*_/*∂β*_*r*_=−*x*_*ir*_*b*^−1^; *∂z*_*i*_/*∂b*=−*z*_*i*_*b*^−1^. The *r*-th and last components of the score functions are given by,

3 $$ \begin{aligned} U_{r}(\boldsymbol{\beta, b})&=-\frac{1}{b}\sum_{i=1}^{n}\Big[\delta_{i} \frac{\partial \log f_{0}(z_{i})}{\partial z_{i}}+(1-\delta_{i})\frac{\partial \log S_{0}(z_{i})}{\partial z_{i}}\Big]x_{ir}, \end{aligned}  $$

4 $$ \begin{aligned} U_{b}(\boldsymbol{\beta, b})&=-\frac{r}{b}-\frac{1}{b}\sum_{i=1}^{n}\left[\delta_{i} \frac{\partial \log f_{0}(z_{i})}{\partial z_{i}}+(1-\delta_{i})\frac{\partial \log S_{0}(z_{i})}{\partial z_{i}}\right]z_{i}. \end{aligned}  $$

The (*p*+2)×(*p*+2) observed information matrix is given by:

$$\begin{aligned} \boldsymbol{I}(\boldsymbol{\beta},b) =& \left(\begin{array}{cc} -\partial^{2}l/\partial\boldsymbol{\beta}\partial\boldsymbol{\beta}^{T} & -\partial^{2}l/\partial\boldsymbol{\beta}\partial b \\ -\partial^{2}l/\partial b\partial\boldsymbol{\beta} & -\partial^{2}l/\partial b^{2} \end{array}\right) \\ =& \frac{1}{b^{2}}\!\left(\!\begin{array}{cc} -\sum_{i=1}^{n}A_{i}\boldsymbol{x}_{i}\boldsymbol{x}_{i}^{T} & -\sum_{i=1}^{n}(A_{i}z_{i} + B_{i})\boldsymbol{x}_{i} \\ \!-\!\sum_{i=1}^{n}(A_{i}z_{i} \!+\! B_{i})\boldsymbol{x}_{i}^{T} &\! -[m \!+\! \sum_{i=1}^{n}(A_{i}z_{i}^{2} +2B_{i} z_{i})] \end{array}\!\right), \end{aligned} $$

where

$$ \begin{aligned} A_{i} &= \delta_{i} \frac{\partial^{2} \log f_{0}(z_{i})}{\partial z_{i}^{2}}+(1-\delta_{i})\frac{\partial^{2} \log S_{0}(z_{i})}{\partial z_{i}^{2}},\\ B_{i} &= \delta_{i} \frac{\partial \log f_{0}(z_{i})}{\partial z_{i}}+(1-\delta_{i})\frac{\partial \log S_{0}(z_{i})}{\partial z_{i}}. \end{aligned} $$

### Firth’s penalized likelihood method for AFT model

In order to remove the first order bias *O*(*n*^−1^) in the MLE of the regression parameter, say ***θ***, of the generalized linear models, [10] introduced a penalized log-likelihood function by adding a penalty term 1/2 log|*I*(***θ***)| to the original log-likelihood function *ℓ*(***θ***). Without loss of generality, Firth’s procedure can be directly applied to the likelihood function of the AFT models given in Eq. (2). For the AFT model (Eq. 1) with a (*p*+2)-dimensional parameter vector ***θ***=(***β***^*T*^,*b*)^*T*^, the penalized log-likelihood function with Firth’s penalty term is given by

5 $$\begin{array}{*{20}l} \ell^{*}(\boldsymbol{\beta},b)=\ell(\boldsymbol{\beta},b)+\frac{1}{2}\log|I (\boldsymbol{\beta},b)|, \end{array} $$

where |*I*(***β***,*b*)|^1/2^ is the Jeffreys invariant prior, whose influence is asymptotically negligible. By adding the penalty term with the original likelihood function given in Eq. (2) one can derive an explicit form of the penalized likelihood function. In addition, according to Firth’s principle, corresponding modified score functions for the *r*-th regression *β*_*r*_ and scale parameter *b* can be written by adding the penalty term in Eqs. (3) and (4) respectively as follows :

6 $$\begin{array}{*{20}l} {U}_{r}^{*}(\boldsymbol{\beta}, b)&={U}_{r}(\boldsymbol{\beta}, b)+\frac{1}{2}\text{tr}\Big[\boldsymbol{I}(\boldsymbol{\beta},b)^{-1}\Big(\frac{\partial \boldsymbol{I}(\boldsymbol{\beta},b)}{\partial \beta_{r}}\Big)\Big]\\ &={U}_{r}(\boldsymbol{\beta}, b)+\frac{\partial} {\partial \beta_{r}} \Big[\frac{1}{2}\log |\boldsymbol{I}(\boldsymbol{\beta},b)| \Big], \end{array} $$

and

7 $$\begin{array}{*{20}l} {U}^{*}_{b}(\boldsymbol{\beta}, b)&={U}_{b} (\boldsymbol{\beta},b)+\frac{1}{2}\text{tr}\Big[\boldsymbol{I}(\boldsymbol{\beta},b)^{-1}\Big(\frac{\partial \boldsymbol{I}(\boldsymbol{\beta},b)}{\partial b}\Big)\Big]\\ &={U}_{b}(\boldsymbol{\beta}, b)+\frac{\partial} {\partial b} \Big[\frac{1}{2}\log |\boldsymbol{I}(\boldsymbol{\beta},b)| \Big]. \end{array} $$

By expanding the above two Eqs. (6–7) one can derive an explicit analytical form of the score equations which ensure finite estimates of both the ***β*** and *b* while solving them using Newton-Raphson method. However, in numerical optimization, the Newton-Raphson’s method can be computationally tedious and inefficient for expansive and complex non-linear problems if the Hessian (second order derivative of the objective function) is directly calculated iteratively. Moreover, Newton-Raphson’s method might not work properly if the Hessian is singular at any iteration. Therefore, rather than solving the modified score equation by the Newton-Raphson algorithm, we directly optimized the penalized likelihood function given in Eq. (7) using a quasi-Newton method referred to the Broyden-Fletcher-Goldfarb-Shanno (BFGS) algorithm, which also ensures finite estimates of both the ***β*** and *b*. It is computationally cheaper and more efficient than the Newton’s method and approximates the Hessian matrix using the gradient (first order derivative of the objective function) at each step rather than iteratively computing it [2]. Thus the penalized likelihood estimates ***θ***^∗^=(***β***^∗^,*b*^∗^) can be obtained from the optimization as follows:

$$\begin{array}{*{20}l} \boldsymbol{\theta}^{*} = \operatorname*{arg\,max}_{\boldsymbol{\beta}^{*},b^{*}} \ell^{*}(\boldsymbol{\beta},b). \end{array} $$

The corresponding standard error of the estimator can be obtained from the approximated Hessian matrix.

### Intercept and scale parameter correction

Firth’s penalized likelihood method is known to reduce first order bias in the estimate of the model parameter by shrinking the estimate towards the true value. However, incorporating the penalty term in the likelihood may cause greater shrinkage of the intercept and scale parameter, which in turn may provides bias in the estimated survival probabilities given by

$$\begin{array}{*{20}l} S(t)=S_{0}\left(\frac{\log t - u(\boldsymbol{x} ; \boldsymbol{\beta}^{*})}{b^{*}}\right)=S_{0}\left(\frac{\log t - \boldsymbol{\beta}^{*T} \boldsymbol{x}}{b^{*}}\right). \end{array} $$

Therefore, a post-hoc adjustment of the intercept and scale parameter estimates in the AFT model has been performed by keeping these parameters out of penalization. The adjustment was performed by following the procedure described in a recent study by [22] for correcting the intercept term in the Firth’s logistic regression. The intercept and scale parameter corrections in AFT models can be administered as follows:

(i) Estimate the coefficients as $\boldsymbol {\hat {\theta }}_{F}=(\hat {\beta }_{F,0},\hat {\beta }_{F,1},\dots,\hat {\beta }_{F,p}, \hat {b}_{F})$ by Firth’s penalization.
(ii) Calculate the linear predictors $\hat {\eta _{i}}=\hat {\beta }_{F,1}x_{i1} + \hat {\beta }_{F,2}x_{i2} + \dots + \hat {\beta }_{F,p}x_{ip}$, omitting the intercept.
(iii) Determine the ML estimate $\hat {\beta }_{0}$ of the intercept and $\hat {b}$ of the scale parameter for the AFT model $Y=\beta _{0}+ \hat {\eta _{i}} + bZ,$ containing only a single predictor $\hat {\eta _{i}}$ with regression coefficient equal to one. This can be achieved by including an offset in a standard procedure or by direct maximum likelihood estimation.
(iv) The resulting estimate $\boldsymbol {\hat {\theta }}_{C} = (\hat {\beta _{0}},\hat {\beta }_{F,1},\dots, \hat {\beta }_{F,p}, \hat {b})$ is then considered as the corrected Firth’s estimate with the intercept and scale parameter replaced by $\hat {\beta _{0}}$ and $\hat {b}$ respectively in the original estimates $\boldsymbol {\hat {\theta }}_{F}$.

This post-hoc adjustment is only required if the interest is to use the model for survival prediction, which is often a primary objective of many studies in clinical prediction research. In the following sub-section, the performance of the Firth’s estimates of the model parameters is investigated using an extensive simulation study and compared the results to those obtained for the standard maximum likelihood techniques.

## Simulation study

### Simulation design

Let us consider survival time *T*_*i*_ for the *i*^*th*^ observation (*i*=1,…,*n*) which follows a probability distribution belonging to log-location scale family of distributions (e.g., Weibull, log-normal and log-logistic) and right censored time *C*_*i*_ which is independent of covariates. We considered two covariates of which one is continuous (*X*_*c*_) and the other is binary (*X*_*b*_). The continuous covariate was generated from a standard normal distribution and the binary covariate was generated from a Bernoulli distribution with probability of event *π*. Then the survival time from the log-location scale family of distributions has been generated as follows:

$$T_{i} = \exp (\beta_{0} + \beta_{c} X_{ic} + \beta_{b} X_{ib} + bZ_{i})$$

where, *β*_0_ denotes the intercept, *β*_*c*_ and *β*_*b*_ represent the regression coefficients associated with the continuous and binary covariates, respectively, *b* is the scale parameter and *Z*_*i*_ is the error term generated from location-scale family of distributions (e.g., Gumbel, normal and logistic distributions). To generate survival times *T*_*i*_ from Weibull, or log-normal or log-logistic distribution (which one we needed), we considered generating *Z*_*i*_ from Gumbel, or Normal or Logistic distribution, respectively. Further the censoring times (*C*_*i*_) were generated independently from the same distribution from where the survival time were generated but by replacing the ***βx***_*i*_ by a constant term, say *λ*, referred to the parameter of the censoring distribution. The value of *λ* control the desired percentage of censoring in the observed data. The observed time-to-event was then defined as *t*_*i*_= min(*T*_*i*_,*C*_*i*_) and the indicator as *δ*_*i*_=*I*(*T*_*i*_≤*C*_*i*_).

Three simulation series were performed separately by considering three different (but widely used) survival distributions such as Weibul, log-normal and log-logistic. Under each distribution (model), the data were generated as described above. The true values of regression and scale parameters were fixed at ***β***=(*β*_0_,*β*_*b*_,*β*_*c*_)=(3,1.2,0.7) and *b*=0.67 respectively for Weibull and log-logistic distribution. A small change was made in the regression parameters ***β***=(*β*_0_,*β*_*b*_,*β*_*c*_)=(1,1.2,0.7) in case of log-normal, considering the same scale parameter. As the data were generated randomly, the percentage of censoring was not exactly the same for all simulated datasets. In order to generate data with a specified censoring proportion,the parameter of the censoring times distribution were determined by iterative algorithm so that the specified censoring proportion would be achieved for the selected parameter values [23]. In the simulation, we reported average of the censoring percentages over 1000 simulations.

For each of the three models, several simulation scenarios were considered by varying the sample size *n* as 30, 50, and 100 and the percentage of censoring as 10, 20, 30, 40, 50, 60, 70, and 80 for each sample size scenario, except for *n*=30 for which it was administered up to 60 because extremely high percentage censoring for very small sample raised serious convergence problem in fitting procedure of the model due to lack of event. Again, fixing the censoring percentage at 20%, we varied the sample size as 15,30,45,60,75,90,105 and 120 to examine the finite sample properties of the estimates under a fixed proportion of censoring.

Further simulation was performed considering a scenario with separation, caused by an influential covariate in the model. In order to create separation, a comparatively larger value of *β*_*b*_ (1.9) was attributed to the binary covariate *X*_*b*_ than the continuous covariate *X*_*c*_ (0.5) to increase its influence so that it can create separation. As discussed by [12], separation have been considered in a survival dataset if, at each failure time, the covariate value for the subjects who were failed is the greatest (or always smallest) among all the all covariate values in the risk set at that time point. It may happen at most failure time points if the influence of the covariate is strong. If separation happens for a binary covariate, according to the definition by Heinze and Schemper, the covariate value separate (fully or partially) the event from non-event (censored) in the data, resulting in a large difference in both survival curves and median survival times between two groups of subjects with respect to covariate values (1/0). Therefore, to explore the existence of separation in a simulated dataset, we tried to mimic the situation by producing a 2×2 contingency table of the censoring status and the binary covariate *X*_*b*_ and we considered as separated data if there is at least one cell with 0 frequency and/or the median survival times between the subjects with *X*_*b*_=0 and those with *X*_*b*_=1 is significantly different with p-value < 0.01. However, not all datasets over the number of simulations have such condition, but a number of datasets have at least one of the cells contains frequency less than or equal to 5 and/or the median survival times between these two groups of subjects is significant with p-value lies between 0.01 and 0.05, which we termed as “near-to-separation" (i.e., partially separated data). The condition of the near-to-separation was discussed in other studies for binary data [19]. We examined the effect of separation or near-to-separation for sample size 50 and censoring percentage 20, 50 and 80.

### Model fitting and evaluating the performance

Under each scenario, we fitted model using both maximum likelihood and Firth’s penalized approaches and evaluated the properties such bias, mean-squared error (MSE) and length of confidence interval (CI). We reported the estimates of the parameters of the model as the average of 1000 simulations. The bias was calculated as the difference between the estimate and true value and the mean squared error as the mean of the squared differences between the estimates in each simulated data and the true value. We also reported both the analytical standard error as the mean of the standard error obtained during model fitting in each simulated data over the number of simulation sets and simulation standard error as the standard deviation of the estimates obtained in each simulation over the number of simulations. All computations were conducted with R statistical software of the version 3.5.3. The standard AFT models with MLE were fitted using the survreg function of the survival package and a self-written function aft.firth was applied to optimize the penalized likelihood function. The R-code of the aft.firth function is available as supplementary document of the article.

### Simulation results

**Fig. 1:**
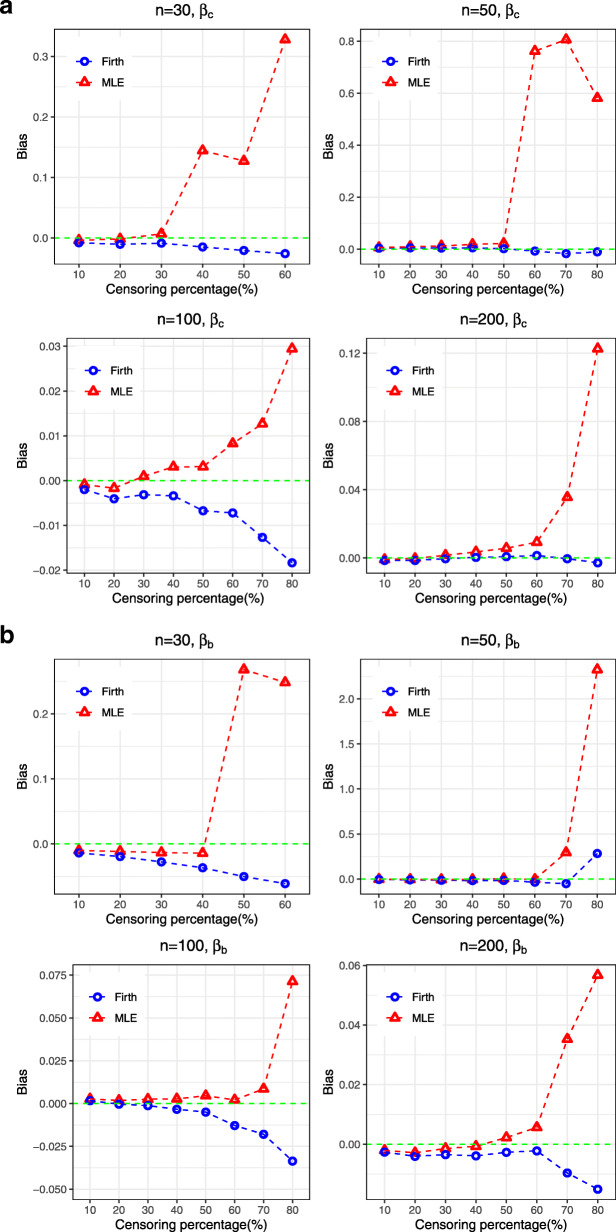
Bias associated with the estimates of regression coefficients (*β*_*c*_ for continuous covariates and *β*_*b*_ for binary covariates) obtained from both MLE and Firth procedure for Weibull AFT model

**Fig. 2:**
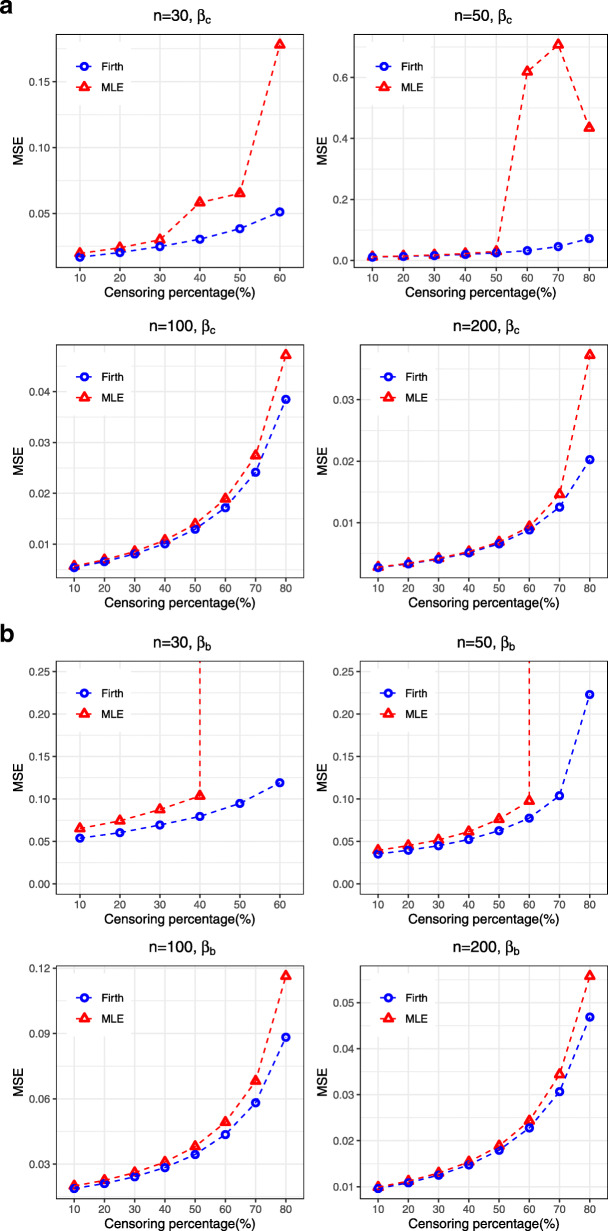
MSE associated with the estimates of regression coefficients (*β*_*c*_ for continuous covariates and *β*_*b*_ for binary covariates) obtained from both MLE and Firth procedure for Weibull AFT model

For the Weibull AFT model, the results suggests that both the coefficients associated with continuous (*β*_*c*_) and binary (*β*_*b*_) covariates are overestimated, in general, by MLE (Table 1). The degree of overestimation increases with the increasing percentage of censored observation. On the contrary, Firth’s penalized method showed some improvements by reducing bias and MSE for the estimates of both *β*_*c*_ and *β*_*b*_ (Figs. 1 and 2). The mean width of Wald based confidence interval is also narrower for Firth’s estimates in all cases, providing more precision in high censored situations. Table 2 shows that the intercept and scale parameter are generally underestimated by the MLE in most cases, except for intercept in high censored cases where it is highly overestimated. After making the post-hoc adjustment to the intercept and scale parameter, the Firth’s penalized method showed improvement by providing estimates relatively closer to the true value.

**Table 1:** Results of both MLE and Firth’s Penalized Likelihood Estimation for both *β*_*b*_ and *β*_*c*_ under Weibull Distribution. Each cell represents mean and standard deviation of estimates over number of valid cases (removing the simulations that were failed to achieve convergence) out of 1000 simulations. The maximum number of convergence failure for MLE is 60 when sample sizze is 50 and censoring rate 80%

			MLE				Firth			
Sample Size (n)	Cens.%	True Coefficients	Estimate	SE	Sim.SE	Width	Estimates	SE	Sim.SE	Width
						(95% CI)				(95% CI)
30	20	*β*_*c*_=1.2	1.198	0.155	0.171	0.606	1.190	0.142	0.170	0.557
	40		1.344	0.193	0.219	0.758	1.185	0.174	0.216	0.681
	60		1.528	0.266	0.334	1.04	1.174	0.225	0.325	0.880
	20	*β*_*b*_=0.7	0.689	0.272	0.307	1.067	0.681	0.245	0.304	0.960
	40		0.686	0.321	0.362	1.259	0.663	0.279	0.350	1.095
	60		0.948	56.262	1.203	220.325	0.639	0.340	0.461	1.332
50	20	*β*_*c*_=1.2	1.210	0.120	0.121	0.469	1.205	0.114	0.120	0.446
	50		1.222	0.170	0.180	0.665	1.202	0.157	0.176	0.616
	80		1.781	0.311	7.080	1.218	1.190	0.268	0.640	1.049
	20	*β*_*b*_=0.7	0.695	0.212	0.224	0.832	0.690	0.199	0.223	0.780
	50		0.704	0.276	0.291	1.082	0.684	0.250	0.284	0.979
	80		3.026	124.560	26.619	486.810	0.983	0.378	8.814	1.482
100	20	*β*_*c*_=1.2	1.200	0.083	0.083	0.325	1.197	0.081	0.082	0.317
	50		1.209	0.117	0.120	0.458	1.199	0.112	0.119	0.440
	80		4.807	0.210	48.926	0.824	1.175	0.193	0.217	0.756
	20	*β*_*b*_=0.7	0.703	0.150	0.150	0.587	0.701	0.145	0.150	0.568
	50		0.708	0.193	0.197	0.758	0.698	0.184	0.194	0.720
	80		3.855	0.327	65.946	1.274	0.668	0.290	0.305	1.137

*β*_*c*_= Coefficient of continuous covariate and *β*_*b*_= Coefficient of binary covariate

**Table 2:** Results of both *β*_0_ and *b* from Maximum Likelihood Estimation and Firth’s Penalized Likelihood Estimation under Weibull Distribution. Each cell represents mean and standard deviation of estimates from valid cases out of 1000 simulations. The maximum number of convergence failure for MLE is 60 when sample sizze is 50 and censoring rate 80%

			MLE			Firth		
Sample Size (n)	Cens.%	True Coefficients	Estimates	SE	Sim.SE	Estimates	SE	Sim.SE
30	20	*β*_0_=3	2.989	0.187	0.213	2.989	0.129	0.212
	40		3.452	0.216	0.246	2.984	0.144	0.242
	60		3.726	0.292	0.354	2.976	0.176	0.333
	20	*b*=0.67	0.618	0.100	0.107	0.616	0.096	0.107
	40		0.607	0.113	0.123	0.603	0.108	0.122
	60		0.597	0.136	0.159	0.585	0.123	0.153
50	20	*β*_0_=3	2.987	0.146	0.154	2.988	0.104	0.153
	50		2.987	0.189	0.197	2.983	0.127	0.194
	80		5.893	6.859	22.755	3.649	0.207	11.591
	20	*b*=0.67	0.642	0.079	0.083	0.641	0.077	0.083
	50		0.635	0.097	0.103	0.631	0.093	0.102
	80		0.598	0.146	0.177	0.584	0.169	0.348
100	20	*β*_0_=3	2.988	0.104	0.107	2.989	0.075	0.107
	50		2.988	0.134	0.139	2.986	0.092	0.137
	80		21.583	0.276	385.896	3.512	0.152	12.415
	20	*b*=0.67	0.654	0.057	0.058	0.653	0.055	0.058
	50		0.649	0.070	0.072	0.647	0.067	0.071
	80		0.633	0.106	0.122	0.626	0.115	0.112

*β*_0_= Intercept and *b*= Scale parameter of the location-scale distribution

**Fig. 3:**
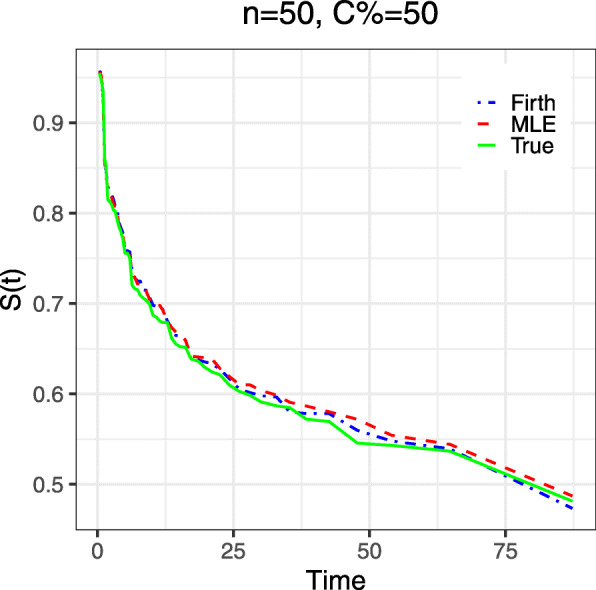
Estimated mean survival probabilities over 500 simulations by both MLE and Firth procedures under Weibull distribution for sample size 30 with censoring percentage C=50%

When the performance of the methods were examined for the scenario with existence of separation, the results depicts that the MLE provides infinitely large estimates in the presence of separation particularly for the regression coefficient (*β*_*b*_) associated with the binary covariate that created separation (Table 3). Conversely, Firth’s penalized method showed significant improvement by providing finite estimates of both the coefficient and its SE. It is notable that the values for MLEs are extremely large for high censoring, whereas the Firth’s procedure succeeds to provide finite estimates in such an extreme case. The amount of improvement is greater for the regression coefficient (*β*_*b*_) associated with the binary covariates than those associated with the continuous covariate. The simulation results for the near-to-separation scenario is similar to that of the separation, but with lower amount of bias. It is also reported that, in the presence of separation, the MLE failed to achieve convergence proving infinitely large value. The rate convergence-failure was 26% when sample size was 50 and censoring was 80%, and the rate decreased to 10% when sample size was 100 with the same level of censoring percentage (results not shown). The convergence-failure rate also decreased with the decreasing censoring percentage and it is very low (often negligible) while there was near-to-separation. In contrast, Firth’s penalized method achieved convergence in all simulation scenarios. Further, the penalized method with ad-hoc adjustment of the intercept and scale parameter outperformed the MLE when it was used for prediction for the survival probabilities (Table 4, Fig. 3). The penalized method provided very close prediction of the true survival probability at the 1st, 2nd, and 3rd quartiles of the survival time (Table 4) and over the whole follow-up time (Fig. 3) in comparison with the MLE.

**Table 3:** Estimates, standard error (SE) and simulation standard error (Sim.SE) of *β*_0_ and *b* from Maximum Likelihood Estimation and Firth’s Penalized Likelihood Estimation under Weibull Distribution in case of separation and near-to separation. Maximum convergence failure by MLE is 26.6% when separation occur over 1000 simulations in case of 80% censoring

			MLE				Firth			
Sample Size (n)	Cens.%	True Coefficients	Estimates	Bias	SE	Sim.SE	Estimates	Bias	SE	Sim.SE
Separation										
50	20	*β*_*c*_=0.5	0.481	-0.019	0.109	0.115	0.479	-0.021	0.103	0.115
	50		0.587	0.087	0.151	0.195	0.575	0.075	0.138	0.184
	80		0.568	0.068	0.259	0.831	0.492	-0.008	0.223	0.397
	20	*β*_*b*_=1.9	1.943	0.043	0.211	0.197	1.928	0.028	0.198	0.195
	50		14.187	12.287	6,559.531	1.566	3.071	1.171	0.879	0.175
	80		104.371	102.471	7020.086	700.557	2.764	0.864	0.813	9.344
Near-to-Separation										
50	20	*β*_*c*_=0.5	0.503	0.003	0.111	0.117	0.500	0.000	0.105	0.117
	50		0.494	-0.006	0.146	0.149	0.485	-0.015	0.134	0.146
	80		1.682	1.182	0.244	23.939	0.484	-0.016	0.203	0.264
	20	*β*_*b*_=1.9	1.904	0.004	0.216	0.229	1.890	-0.010	0.202	0.227
	50		2.030	0.130	0.354	0.348	1.934	0.034	0.309	0.315
	80		2.855	0.955	0.636	21.649	1.433	-0.467	0.468	0.459

*β*_*c*_= Coefficient of continuous covariate and *β*_*b*_= Coefficient of binary covariate

**Table 4:** Estimates of survival probabilities (mean over 500 simulations) at the 1^*st*^, 2^*nd*^ and 3^*rd*^ quantile of survival times of Weibull distribution with different values of binary covariates and the mean value of continous covariate for sample size 50 and censoring 50%

Binary covariate	Quartiles	True	MLE	Firth
*X*_2_=0	1st	0.750	0.780	0.769
	2nd	0.500	0.543	0.529
	3rd	0.250	0.294	0.278
*X*_2_=1	1st	0.750	0.757	0.751
	2nd	0.500	0.498	0.499
	3rd	0.250	0.247	0.249

**Fig. 4:**
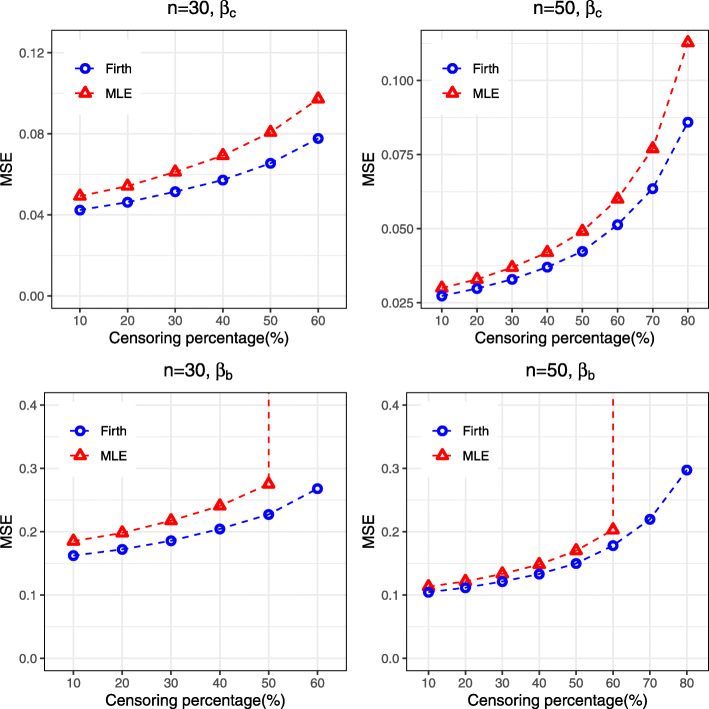
MSE associated with the estimates of regression coefficients (*β*_*c*_ for continuous covariates and *β*_*b*_ for binary covariates) obtained from both MLE and Firth procedure for Log-logistic AFT model

Similar findings were found for the log-logistic AFT model, where Firth’s penalized method showed improvement over the MLE by reducing bias and MSE and providing narrower confidence interval, particularly when censoring percentage is high, for both the regression coefficients *β*_*c*_ and *β*_*b*_ in the model (Supplementary Table S1, Fig. 4). Similarly, for the intercept (*β*_0_) and scale parameter (*b*), Firth’s method with ad-hoc correction showed better performance than the MLE, particularly for high censoring situation (Supplementary Table S2). The correction procedure renders better performance in rare event situations under log-logistic distribution. In the presence of separation, Firth’s penalized method outperforms the MLE by reducing bias to some extent and providing narrower confidence interval (Supplementary Table S3). Similar findings can also be observed for survival prediction for the log-logistic AFT model (results not shown).

**Fig. 5:**
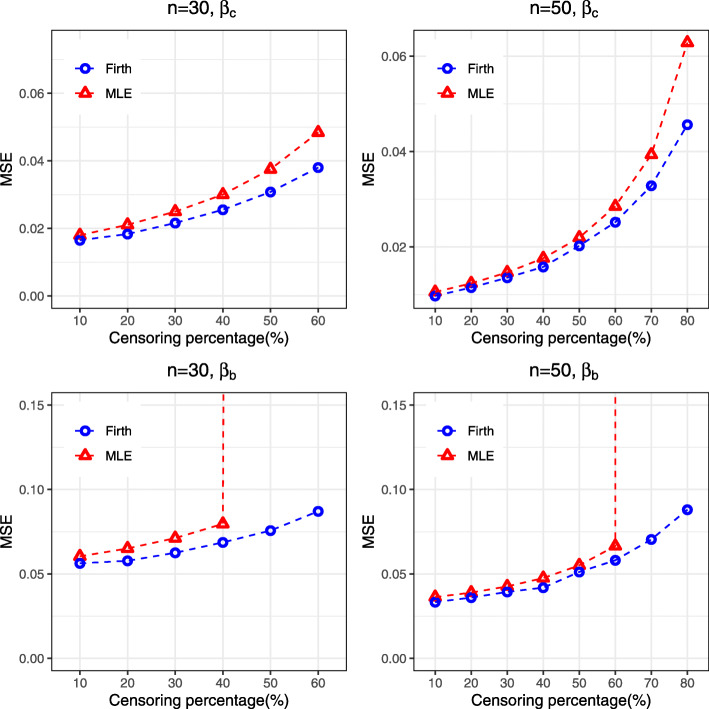
MSE associated with the estimates of regression coefficients (*β*_*c*_ for continuous covariates and *β*_*b*_ for binary covariates) obtained from both MLE and Firth procedure for Log-normal AFT model

For the log-normal AFT model, Firth’s penalized method also showed similar performance by providing lower MSE and narrower confidence intervals than MLE (Supplementary Table S4, Fig. 5). The amount of improvement by Firth’s penalized method is also greater for the regression coefficient (*β*_*b*_) associated with binary covariates. For the intercept and scale parameters, the correction of Firth’s procedure provides an improvement over the MLE (Supplementary Table S5). Similarly, greater performance were also achieved by the Firth’s penalized method when it was used for prediction of the survival probabilities (results not shown).

## Illustration using prostate cancer data

The methods are illustrated using prostate cancer data, which were previously used in a study by [5] and are publicly accessible (https://hbiostat.org/data/). They conducted an exploratory analysis on the data from a clinical trial of estrogen treatment for a total of 502 prostatic cancer patients several survival status (1 - alive, 2 - dead from prostatic cancer, 3 - dead from heart or vascular disease, 4 - dead from cerebrovascular accident, 5 - dead from pulmonary embolus, 6 - dead from other cancer, 7 - dead from respiratory disease, 8 - dead from other specific non-cancer cause, 9 - dead from other unspecified non-cancer cause and 10 - dead from unknown cause). In this study, we firstly focus on the effectiveness of the treatment on the survival of patient with prostatic cancer and hence observe the time-to-death due to prostatic cancer or not (censoring percentage 53.82). An exploratory analysis with a 2×2 contingency table showed that the original data does not suffer from the problem of separation. Therefore, for an illustrative purpose of the method discussed, a random sub-sample of patients who are either alive or died from prostatic cancer was taken from the original data in order to create separation and near-to-separation. Furthermore, since the number of patients who died from respiratory disease and pulmonery embolus were very small in the original data (16 and 14 respectively) making these events rare, we also considered two more scenarios with the data: one with patients who are alive or died from respiratory disease and another with patients who are alive or died from pulmonery embolus. Each of these two scenarios created near-to-separation (non-zero cells with few observations).

The covariates of interest are: treatment (0, low; 1, high-dose), age (0, < 75 years; 1, 75 to 80 years; 2, ≥ 80 years), weight index caluctaed as weight (kg) - height(cm) + 200 (0, ≥ 100; 1, 80-99; 2, < 80), performance rating (0, normal; 1, limitation of activity), history of cardiovascular disease (0, no; 1, yes), serum haemoglobin (0, ≥ 12g/100 ml; 1, 9-12g/100 ml; 2, < 9g/100 ml), size of primary lesion (0, < 30 *cm*^2^; 1, ≥ 30 *cm*^2^), and Gleason stage/grade category (0, ≥ 10; 1, > 10). The variables are denoted as AG (patient age), WT(weight index), PF(performance rating), HX (history of cardiovascular disease), HG (serum haemoglobin), SZ (size of primary lesion) and SG (Geason stage/grade category). For each scenario, we considered a Weibull AFT model with the covariates selected based on the analysis discussed in literature [5, 14].

$$\begin{aligned} \log (T_{i}) &= \beta_{0} + \beta_{1} Treatment_{i} + \beta_{2} AG_{i} + \beta_{3} WT_{i} + \beta_{4} PF_{i} +\\&\beta_{5} HX_{i}+ \beta_{6} HG_{i} +\beta_{7} SZ_{i} +\beta_{8} SG_{i} + bz_{i},\! \quad i=1\dots, n. \end{aligned} $$

The above Weibull AFT model was fitted for both the cases of separation and near-to separation with patients died from prostatic cancer and the near-to separation case with patients died from respiratory disease and pulmonary embolus.

### Analysis and results of prostate cancer data

As mentioned, for illustrative purpose, a random sample of size 30 was taken from the original sample to create separation and near-to-separation in the data, respectively. A 2×2 contingency table (Table 5) between the estrogen treatment and patient status shows two different forms of separation. Moreover, patient’s age and haemoglobin level have also created separation in the outcome variable. Table 6 reveals that the MLE of regression coefficient of the treatment status responsible to create separation in the sub-sample is very large comparative to that of the Firth’s estimate of the coefficient making MLE uninterpretable. Under the separation scenario, the MLE fails to deliver a standard error for the regression coefficient of age resulting in a disrupted inference. Conversely, Firth’s procedure produces a finite estimate and standard error for the corresponding covariate. Although, the MLE becomes smaller with increased degrees of overlapping in the near-to-separation for the treatment status, the Firth’s procedure provides both smaller estimate and standard error for this covariate. In terms of standard error, the penalized estimates are more efficient than MLE in each separation scenarios.

**Table 5:** Contingency tables between dichotomous covariates (treatment) and response (prostate cancer status) showing separation and near-to-separation

	Separation		Near-to-Separation	
	Status		Status	
Treatment	alive(0)	dead(1)	alive(0)	dead(1)
low-dose(0)	12	8	9	6
high - dose (1)	10	0	12	3
	Status		Status	
Age	alive(0)	dead(1)	alive(0)	dead(1)
≤ 75 years(0)	17	8	14	7
75-80g/ 100 ml(1)	5	0	6	2
≥ 80g/ 100 ml(2)	−−	−−	1	0
	Status		Status	
Serum haemoglobin (HG)	alive(0)	dead(1)	alive(0)	dead(1)
≥ 12g/ 100 ml(0)	20	7	18	9
9-12g/ 100 ml(1)	2	0	3	0
< 9g/ 100 ml(2)	0	1	−−	−−

**Table 6:** Estimates of regression parameters and their standard error obtained from MLE and Firth’s procedure by fitting Weibull AFT model for prostate cancer data under separation and near-to-separation

	Separation				Near-to-Separation			
	MLE		Firth		MLE		Firth	
Predictors	Estimates	SE	Estimates	SE	Estimates	SE	Estimates	SE
Treatment	11.842	9,561.118	1.129	0.409	1.146	0.600	0.846	0.271
Age	11.475	0.00	1.013	0.428	0.380	0.804	0.132	0.565
WT	0.309	0.502	− 0.112	0.199	− 1.316	0.597	− 0.598	0.213
PF	-0.895	0.888	-0.981	0.388	-1.138	1.417	-0.854	0.720
HX	0.534	0.615	0.503	0.251	1.547	1.057	0.905	0.616
HG	-1.141	0.731	-1.063	0.313	13.101	7,825.785	1.587	0.684
SZ	-0.537	0.778	-0.054	0.259	0.506	0.918	0.389	0.591
SG	-1.966	0.872	-0.321	0.092	-2.071	0.743	-0.345	0.080
Intercept	5.218	0.728	7.671	0.181	5.924	1.006	8.171	0.252
scale (b)	0.518	0.182	0.398	0.096	0.694	0.219	0.608	0.145

WT = weight index, PF= performance rating, HX= history of cardiovascular disease, HG= serum haemoglobin, SZ= size of primary lesion, SG= Gleason stage/grade category

### Analysis and results of respiratory disease and pulmonary embolus

The contingency Table 7 between the estrogen treatment and patient’s survival status (death from respiratory disease and pulmonary embolus) shows the existence of near-to-separation in the data. Furthermore, performance rating, serum haemoglobin level and size of primary lesion have also created separation in outcome variable in both cases of respiratory disease and pulmonary embolus. Here, total sample size consists of 148 alive with only 16 (Respiratory disease) and 14 (Pulmonary embolus) events or failure and near-to-separation can be observed. The censoring percentage is 91.92% (Respiratory disease) and 93.08% (Pulmonary embolus) respectively. The Table 8 reveals that similar to the scenario with prostatic cancer, the Firth’s estimates of coefficient of treatment are smaller in magnitude and have smaller standard error than MLE in each case. Moreover, zero or extremely large standard errors estimated by MLE for some covariates (performance rating, serum haemoglobin and size of primary lesion) indicate convergence failure during estimation. In contrary, Firth’s procedure succeeds to deliver finite estimates and smaller standard error for all covariates in each case.

**Table 7:** Contingency tables between dichotomous covariates (treatment) and response (respiratory disease and pulmonary embolus status)

	Respiratory disease		Pulmonary embolus	
	Status		Status	
Treatment	alive(0)	dead(1)	alive(0)	dead(1)
low-dose(0)	61	10	61	4
high - dose (1)	87	6	87	10
	Status		Status	
Performance rating (PF)	alive(0)	dead(1)	alive(0)	dead(1)
normal(0)	142	16	142	12
limitation of activity(1)	6	0	6	2
	Status		Status	
Serum haemoglobin (HG)	alive(0)	dead(1)	alive(0)	dead(1)
≥ 12g/ 100 ml(0)	131	15	131	11
9-12g/ 100 ml(1)	16	1	16	3
< 9g/ 100 ml(2)	1	0	1	0
	Status		Status	
Size of primary lesion (SZ)	alive(0)	dead(1)	alive(0)	dead(1)
< 30 cm^2^(0)	141	15	141	12
≥ 30 cm^2^(1)	5	1	5	2

**Table 8:** Estimates of regression parameters and their standard error obtained from MLE and Firth’s procedure by fitting Weibull AFT model for time-to-event data with outcome both respiratory disease and pulmonary embolus

	Respiratory disease				Pulmonary embolus			
	MLE		Firth		MLE		Firth	
Predictors	Estimates	SE	Estimates	SE	Estimates	SE	Estimates	SE
Treatment	0.204	0.478	0.124	0.284	-0.588	1.086	-0.292	0.575
Age	-1.131	0.419	-0.755	0.243	-1.481	0.900	-0.913	0.471
WT	-0.535	0.351	-0.307	0.202	0.218	0.821	0.070	0.445
PF	14.193	*∞*	-0.259	0.750	26.677	*∞*	-0.676	1.518
HX	0.001	0.488	− 0.037	0.289	-0.646	1.121	− 0.463	0.625
HG	14.733	6,113.595	0.857	0.751	− 0.672	1.284	− 0.585	0.672
SZ	14.605	*∞*	− 0.309	0.731	− 3.777	1.697	− 2.472	0.895
SG	0.083	0.463	0.025	0.076	-0.503	1.077	− 0.029	0.146
Intercept	6.772	0.904	5.931	0.336	10.031	2.075	8.942	0.969
scale (b)	0.779	0.262	0.646	0.073	1.657	3.438	1.423	0.159

AG = age, WT = weight index, PF= performance rating, HX= history of cardiovascular disease, HG= serum haemoglobin, SZ= size of primary lesion, SG= Gleason stage/grade category

## Discussion

The AFT model is being widely used to analyze survival data from health and reliability engineering because of its intuitive interpretation connecting directly with failure time. For the rare event survival data or data with small in size, separation or monotone likelihood often exists in the fitting process of the AFT model using maximum likelihood estimation technique. The paper investigated the performance of the MLE of the AFT model in such data condition and addressed these issues by introducing a penalized likelihood approach by adding a Firth-type penalty term to the original likelihood. Further a post-hoc correction was made by keeping the intercept and scale parameter out of penalization to improve the estimates of predicted survival probabilities. The performance of the proposed method was evaluated using an extensive simulation study considering AFT model under three different (but widely used) distributions of the log-location scale family separately. For each of the models, the proposed penalized method has been shown to provide superior performance over MLE by solving the problem of monotone likelihood reflected by achieving convergence and providing estimates with lower bias and MSE and narrower confidence interval, in most simulation scenarios.

In particular, when sample size is small and/or percentage of censoring is high, the regression coefficient estimates (both binary and continuous) from penalized likelihood are generally shown to have lower bias and MSE with narrower confidence interval than that for MLE. Again, in the presence of any form of separation, the simulation results revealed that the MLE provided large amount of bias and MSE (often infinitely large value indicating frequent convergence failure) for the estimates of the regression coefficient, particularly those associated with the binary covariates that created separation. On the contrary, the penalized method showed improvement over MLE by achieving convergence and reducing bias and MSE to some extent and providing narrower confidence interval. However, comparable results are observed for both methods for the regression coefficient associated with continuous covariates that didn’t make separation. Simulation study also showed that the performance of the penalized likelihood estimation tends to be better than the MLE in separation than that for near-to-separation, indicating the effectiveness of the proposed method in extreme situations of separation. Furthermore, the post-hoc adjustment of the intercept and scale parameters under the penalized method has been shown to generate improved intercept and scale parameter estimates over MLEs by lowering the bias and consequently to provide relatively accurate estimates of the survival probabilities at different quartiles of the survival times. Simulation results of this study are quite similar to those with the other studies in the recent years which discussed Firth-type penalized estimates of regression models such as logistic regression [13, 22] and Cox regression [12].

An illustration of the methods using prostate cancer data supported the simulation findings by providing estimates with intuitive interpretation. However, demonstration of a rigorous application of this approach to a data with existence of high rate of censoring and/or existence of separation was not possible here to due to lack of access to such data, which may be useful for the practical users of this method. The proposed penalized method for AFT model underestimated the true SE for some scenarios (small smaple with high rate of censoring) and hence provided biased estimate of confidence interval, hence further study may be required with profile likelhood based confidence interval to address this problem. Further study may also be required to compare the performance of the Firth’s penalization for the Cox proportional hazard and AFT medals to address such problems related to small sample, high censoring and separation, because of a physical difference between of theses two models. In addition, one may compare the performance of Firth’s penalized AFT model with other penalized methods such as Ridge regression, LASSO etc.

## Conclusion

The findings of the paper suggest that if the sample size is small and/or the percentage of censoring is high, the performance of MLE becomes unreliable as it provides biased estimates and creates separation leading to monotone likelihood with frequent convergence failure. The proposed penalized approach showed superior performance over MLE by reducing bias and MSE and solving the problem of separation. Therefore, if sample size is relatively small (e.g., *n*≤50) or there is evidence of high censoring and/or separation in the data, it is recommended to apply Firth’s penalized method for fitting AFT models.

## Supplementary Information

**Additional file 1** Supplementary Tables.

## Data Availability

The dataset used in this study can be downloaded freely from the public domain, under the authority of the department of Biostatistics, Vanderbilt University, USA, at https://hbiostat.org/data/.

## References

[1] Albert A, Anderson JA (1984). On the existence of maximum likelihood estimates in logistic regression models. Biometrika.

[2] Arora JS. Introduction to optimum design. 4th ed: Elsevier; 2004.

[3] Bryson MC, Johnson ME (1981). The incidence of monotone likelihood in the cox model. Technometrics.

[4] Bull SB, Mak C, Greenwood CM (2002). A modified score function estimator for multinomial logistic regression in small samples. Comput Stat Data Anal.

[5] Byar D, Green S (1980). The choice of treatment for cancer patients based on covariate information. Bull Cancer.

[6] Cordeiro GM, Cribari-Neto F (1998). On bias reduction in exponential and non-exponential family regression models. Commun Stat-Simul Comput.

[7] Cordeiro GM, McCullagh P (1991). Bias correction in generalized linear models. J R Stat Soc Ser B Methodol.

[8] Cox DR (1972). Regression models and life-tables. J R Stat Soc Ser B Methodol.

[9] Cox DR, Oakes D. Analysis of survival data. 1st ed: Chapman and Hall/CRC; 1984.

[10] Firth D (1993). Bias reduction of maximum likelihood estimate. Biometrika.

[11] Hauck Jr WW, Donner A (1977). Wald’s test as applied to hypotheses in logit analysis. J Am Stat Assoc.

[12] Heinze G, Schemper M (2001). A solution to the problem of monotone likelihood in cox regression. Biometrics.

[13] Heinze G, Schemper M (2002). A solution to the problem of separation in logistic regression. Stat Med.

[14] Kay R (1986). Treatment effects in competing-risks analysis of prostate cancer data. Biometrics.

[15] Kosmidis I, Firth D (2011). Multinomial logit bias reduction via the Poisson log-linear model. Biometrika.

[16] Lawless JF. Statistical models and methods for lifetime data. 2nd ed: John Wiley & Sons, Inc; 2011.

[17] Lesaffre E, Albert A (1989). Partial separation in logistic discrimination. J R Stat Soc Ser B Methodol.

[18] Leung DH-Y, Wang Y-G (1998). Bias reduction using stochastic approximation. Aust N Z J Stat.

[19] Mondal M, Rahman MS (2019). Bias-reduced and separation-proof GEE with small or sparse longitudinal binary data. Stat Med.

[20] Oakes D (1977). The asymptotic information in censored survival data. Biometrika.

[21] Pettitt A, Kelly J, Gao J (1998). Bias correction for censored data with exponential lifetimes. Stat Sin.

[22] Puhr R, Heinze G, Nold M, Lusa L, Geroldinger A (2017). Firth’s logistic regression with rare events: accurate effect estimates and predictions. Stat Med.

[23] Qian J, Li B, Chen P-y (2010). Generating survival data in the simulation studies of cox model. 2010 Third International Conference on Information and Computing.

[24] Schaefer RL (1983). Bias correction in maximum likelihood logistic regression. Stat Med.

[25] Vaeth M (1985). On the use of wald’s test in exponential families. Int Stat Rev/Rev Int Stat.

