# Supplementary material for: On estimation for accelerated failure time models with small or rare event survival data
Source: BMC Med Res Methodol. 2022 Jun 11;22:169. doi: 10.1186/s12874-022-01638-1 (PMC9188212; doi:10.1186/s12874-022-01638-1)
Supplement: Supplementary file 1 — Additional file 1 Supplementary Tables. [file 12874_2022_1638_MOESM1_ESM.pdf]

Supplementary Tables

Table S1: Estimates, standard error (SE), simulation standard error (Sim.SE) and length of 95% confidence intervals (CIs) of β_c and β_b from maximum likelihood estimation and Firth's penalized likelihood estimation under log-logistic distribution. Each cell represents mean and standard deviation of estimates from 1000 samples. The maximum Monte Carlo Error is 0.062.

			MLE				Firth			
Sample Size (n)	Cens.%	True Coefficients	Estimates	SE	Sim.SE	Width (95% CI)	Estimates	SE	Sim.SE	Width (95% CI)
30	20	$\beta_c = 1.2$	1.202	0.233	0.253	0.913	1.193	0.215	0.253	0.843
	40		1.210	0.263	0.293	1.031	1.194	0.239	0.291	0.937
	60		1.223	0.311	0.371	1.219	1.195	0.279	0.368	1.093
	20	$\beta_b = 0.7$	0.701	0.445	0.472	1.745	0.699	0.415	0.472	1.626
	40		0.697	0.491	0.533	1.923	0.690	0.452	0.529	1.772
	60		0.713	51.148	1.116	200.502	0.673	0.517	0.638	2.027
50	20	$\beta_c = 1.2$	1.210	0.181	0.188	0.710	1.204	0.173	0.188	0.677
	50		1.217	0.221	0.238	0.866	1.201	0.206	0.236	0.807
	80		1.241	0.333	0.399	1.306	1.202	0.293	0.392	1.149
	20	$\beta_b = 0.7$	0.693	0.349	0.365	1.366	0.690	0.334	0.366	1.309
	50		0.698	0.412	0.433	1.617	0.689	0.387	0.429	1.517
	80		0.815	91.545	1.949	358.137	0.674	0.545	0.705	2.136
100	20	$\beta_c = 1.2$	1.208	0.126	0.127	0.495	1.205	0.123	0.126	0.482
	50		1.209	0.154	0.160	0.602	1.201	0.148	0.159	0.582
	80		1.216	0.231	0.243	0.907	1.198	0.218	0.241	0.854
	20	$\beta_b = 0.7$	0.698	0.246	0.248	0.963	0.696	0.240	0.247	0.941
	50		0.688	0.291	0.300	1.141	0.684	0.282	0.300	1.106
	80		0.701	7.703	0.631	30.196	0.676	0.415	0.463	1.626

β_c = Coefficient of continuous covariate and β_b = Coefficient of binary covariate

Table S2: Estimates, standard error (SE) and simulation standard error (Sim.SE) of β_0 and b from maximum likelihood estimation and Firth's Penalized Likelihood Estimation under log-logistic distribution. Each cell represents mean and standard deviation of estimates from 1000 samples.

Sample Size (n)	Cens.%	True Coefficients	MLE Estimates	MLE SE	MLE Sim.SE	Firth Estimates	Firth SE	Firth Sim.SE
30	20	$\beta_0 = 3$	2.995	0.308	0.313	2.994	0.212	0.312
	40		2.999	0.338	0.368	2.996	0.232	0.364
	60		3.027	10.364	0.611	3.001	0.267	0.453
	20	$b = 0.67$	0.626	0.106	0.111	0.625	0.105	0.111
	40		0.612	0.118	0.127	0.611	0.116	0.127
	60		0.585	0.136	0.152	0.583	0.129	0.151
50	20	$\beta_0 = 3$	3.006	0.242	0.255	3.007	0.170	0.255
	50		3.005	0.285	0.303	3.003	0.200	0.301
	80		3.044	17.442	0.766	3.000	0.290	0.539
	20	$b = 0.67$	0.647	0.085	0.085	0.647	0.084	0.085
	50		0.635	0.102	0.108	0.634	0.099	0.107
	80		0.585	0.143	0.167	0.581	0.130	0.165
100	20	$\beta_0 = 3$	2.999	0.171	0.172	2.999	0.121	0.172
	50		3.005	0.202	0.205	3.004	0.145	0.205
	80		3.011	0.316	0.338	3.004	0.222	0.334
	20	$b = 0.67$	0.655	0.060	0.062	0.654	0.060	0.062
	50		0.649	0.073	0.079	0.648	0.072	0.078
	80		0.633	0.108	0.117	0.631	0.099	0.117

β_0 = Intercept and b = Scale parameter of the location-scale distribution

Table S3: Estimates, standard error (SE) and simulation standard error (Sim.SE) of β_0 and b from maximum likelihood estimation and Firth's penalized likelihood estimation under log-logistic distribution in case of separation and near-to separation.

Separation

Sample Size (n)	Cens.%	True Coefficients	MLE Estimates	MLE Bias	MLE SE	MLE Sim.SE	Firth Estimates	Firth Bias	Firth SE	Firth Sim.SE
50	20.0	$\beta_c = 0.5$	0.497	-0.003	0.171	0.180	0.495	-0.005	0.163	0.179
	50.0		0.460	-0.040	0.199	0.266	0.465	-0.035	0.189	0.266
	80.0		0.416	-0.084	0.368	0.348	0.373	-0.127	0.278	0.338
	20.0	$\beta_b = 1.9$	1.977	0.077	0.366	0.396	1.968	0.068	0.350	0.397
	50.0		2.068	0.168	0.381	0.694	2.044	0.144	0.357	0.681
	80.0		702.734	700.834	$7,453.466$	$2,207.609$	1.709	-0.191	0.800	1.263

Near to Separation

Sample Size (n)	Cens.%	True Coefficients	MLE Estimates	MLE Bias	MLE SE	MLE Sim.SE	Firth Estimates	Firth Bias	Firth SE	Firth Sim.SE
50	20.0	$\beta_c = 0.5$	0.499	-0.001	0.175	0.177	0.497	-0.003	0.167	0.177
	50.0		0.498	-0.002	0.204	0.217	0.492	-0.008	0.191	0.215
	80.0		0.516	0.016	0.318	0.359	0.499	-0.001	0.268	0.351
	20.0	$\beta_b = 1.9$	1.899	-0.001	0.371	0.384	1.896	-0.004	0.355	0.383
	50.0		1.951	0.051	0.418	0.440	1.933	0.033	0.392	0.435
	80.0		1.921	0.021	0.608	0.693	1.877	-0.023	0.527	0.671

β_c = Coefficient of continuous covariate and β_b = Coefficient of binary covariate

Table S4: Estimates, standard error (SE), simulation standard error (Sim.SE) and length of 95% confidence intervals (CIs) of β_c and β_b from Maximum Likelihood Estimation and Firth's Penalized likelihood estimation under log-normal Distribution. Each cell represents mean and standard deviation of estimates from 1000 samples. The maximum Monte Carlo Error is 0.022.

Sample Size (n)	Cens.%	True Coefficients	MLE				Firth			
			Estimates	SE	Sim.SE	Width (95% CI)	Estimates	SE	Sim.SE	Width (95% CI)
30	20	$\beta_c = 1.2$	1.210	0.145	0.154	0.568	1.196	0.135	0.151	0.531
	40		1.211	0.173	0.187	0.678	1.178	0.158	0.182	0.621
	60		1.220	0.219	0.257	0.859	1.159	0.191	0.247	0.747
	20	$\beta_b = 0.7$	0.707	0.255	0.273	1.000	0.699	0.240	0.270	0.942
	40		0.708	0.282	0.310	1.106	0.691	0.262	0.301	1.027
	60		0.728	11.206	0.478	43.883	0.683	0.295	0.357	1.155
50	20	$\beta_c = 1.2$	1.196	0.111	0.119	0.436	1.187	0.106	0.119	0.417
	50		1.199	0.148	0.158	0.581	1.172	0.140	0.156	0.547
	80		1.222	0.250	0.286	0.979	1.131	0.202	0.259	0.792
	20	$\beta_b = 0.7$	0.700	0.197	0.205	0.773	0.695	0.190	0.203	0.743
	50		0.696	0.235	0.249	0.920	0.681	0.225	0.244	0.883
	80		0.783	45.379	0.697	177.886	0.668	0.295	0.381	1.156
100	20	$\beta_c = 1.2$	1.199	0.079	0.079	0.308	1.194	0.076	0.079	0.300
	50		1.201	0.104	0.109	0.408	1.187	0.099	0.108	0.390
	80		1.215	0.172	0.188	0.673	1.168	0.159	0.184	0.624
	20	$\beta_b = 0.7$	0.693	0.141	0.144	0.553	0.691	0.137	0.143	0.539
	50		0.697	0.167	0.175	0.655	0.688	0.161	0.173	0.630
	80		0.710	0.249	0.264	0.975	0.679	0.235	0.253	0.923

β_c = Coefficient of continuous covariate and β_b = Coefficient of binary covariate

Table S5: Estimates, standard error (SE), simulation standard error (Sim.SE) of β_0 and b from maximum likelihood Estimation and Firth's Penalized likelihood estimation under log-normal distribution. Each cell represents mean and standard deviation of estimates from 1000 samples.

Sample Size (n)	Cens.%	True Coefficients	MLE Estimates	MLE SE	MLE Sim.SE	Firth Estimates	Firth SE	Firth Sim.SE
30	20	$\beta_0 = 1$	0.997	0.175	0.192	0.998	0.122	0.191
	40		0.997	0.194	0.219	0.992	0.134	0.214
	60		1.006	0.242	0.282	0.977	0.155	0.262
	20	$b = 0.67$	0.627	0.091	0.099	0.626	0.090	0.099
	40		0.616	0.104	0.114	0.612	0.098	0.112
	60		0.594	0.124	0.141	0.586	0.109	0.136
50	20	$\beta_0 = 1$	0.997	0.136	0.142	0.998	0.096	0.141
	50		1.001	0.165	0.175	0.994	0.115	0.171
	80		1.022	2.009	0.375	0.946	0.167	0.321
	20	$b = 0.67$	0.639	0.072	0.075	0.638	0.071	0.074
	50		0.629	0.090	0.096	0.626	0.083	0.094
	80		0.581	0.134	0.153	0.567	0.106	0.145
100	20	$\beta_0 = 1$	1.005	0.097	0.098	1.005	0.070	0.098
	50		1.005	0.118	0.124	1.001	0.083	0.122
	80		1.014	0.218	0.229	0.976	0.125	0.215
	20	$b = 0.67$	0.655	0.052	0.053	0.655	0.051	0.053
	50		0.648	0.065	0.069	0.646	0.061	0.068
	80		0.623	0.099	0.107	0.615	0.080	0.103

β_0 = Intercept and b = Scale parameter of the location-scale distribution distribution
